# Supplementary material for: Dispositional optimism weakly predicts upward, rather than downward, counterfactual thinking: A prospective correlational study using episodic recall
Source: PLoS One. 2020 Aug 14;15(8):e0237644. doi: 10.1371/journal.pone.0237644 (PMC7428155; doi:10.1371/journal.pone.0237644)
Supplement: S1 Appendix — A summary table of nine studies across six papers published between 1995 and 2015, which indicate that optimism predicts downward (vs. upward) counterfactual thinking. This summary table reports the authors and year of publication; the study number within the publication (if applicable); the total sample size of the study (if reported); the design of that study including the conditions participants were assigned to or whether the design was correlational; how counterfactuals were elicited (i.e., in response to what prompts or events); the scale that was used to capture trait optimism; what optimism was compared to (if applicable); and, how counterfactuals were classified as downward or upward. (DOCX) [file pone.0237644.s001.docx]

# Appendix

# S1. Summary Table of Prior Research
