# Supplementary material for: Dispositional optimism weakly predicts upward, rather than downward, counterfactual thinking: A prospective correlational study using episodic recall
Source: PLoS One. 2020 Aug 14;15(8):e0237644. doi: 10.1371/journal.pone.0237644 (PMC7428155; doi:10.1371/journal.pone.0237644)
Supplement: S1 File — (DOCX) [file pone.0237644.s002.docx]

**Online Supplemental Materials**

**Meta-analysis R code**

install.packages("metafor")

library(metafor)

r.to.z <- function(r){ (1/2) * log((1+r)/(1-r)) }

z.to.r <- function(z){ (exp(2*z)-1) / (exp(2*z)+1) }

rr <- c(0.0987, 0.0413, -0.0333, 0.0633, 0.1268, 0.0626, 0.0677)

nn <- c(197, 494, 199, 290, 196, 525, 1149)

zz <- r.to.z(rr)

vv <- 1/(nn-3)

mm <- rma.uni(zz, vv)

mm

z.to.r(mm$b)

**Preliminary studies—P1 through P6**

**Study P1**

Study P1 was a first test of whether and how dispositional optimism relates to counterfactual direction of comparison. In order to introduce variability in the tendency to report upward and downward counterfactuals, participants reported counterfactual alternatives to autobiographical events that were either positively or negatively valanced. This outcome valence manipulation derived from past demonstrations that upward counterfactuals are more common following negative outcomes whereas downward counterfactuals are more common following positive outcomes [40]. Participants answered questions designed to assess counterfactual direction, then completed the LOT-R as a measure of optimism. The key question was whether optimism would predict relatively greater upward counterfactual thinking (as our theoretical perspective suggests) versus relatively greater downward counterfactual thinking (as the extant literature suggests).

**Method**

All analyses for preliminary studies were conducted using JMP Pro v14.1.0, unless otherwise specified. Participants were two hundred and one adults drawn from Mechanical Turk (MTurk) (65% female; *M*_age_ = 34.4, SD_age_ = 11.3). Informed consent was obtained by all participants in this, and all subsequent studies, prior to data collection. An independent coder, blind to our hypotheses, was employed to assess whether participants followed the instructions to the initial study prompts (i.e., recalling an event and generating a counterfactual). Any participants not following these instructions, as determined by this independent coder, were excluded from analyses. Accordingly, four participants were excluded (e.g., answered “na” to the counterfactual prompt), leaving a final sample of *N* = 197. The study ran from 2-27-2017 to 3-1-2017.

The study employed a 2 (outcome valence: positive vs. negative) × continuous (optimism) between-subject design. Upon entering the study, all participants read the instructions, “This survey asks you about events from your recent past. Your job is to answer brief questions about what you remember.” Participants were randomly assigned to the outcome valence condition using the following prompt: “Think back to a recent POSITIVE [NEGATIVE] experience. In the space below, please share a few details about this experience.” Next, we elicited counterfactuals via the prompt: “After having experiences like this, sometimes people have thoughts like ‘what if’ - in that they think about how things could have gone differently. In the space below, please share one ‘what if’ thought.”

Participants responded to a three-item rating measure designed to assess the direction of the counterfactual they reported: (a) “Does your ‘what if’ thought focus more on how things could have gone better or how things could have gone worse?”; (b) “In general when you look back at this experience, do you tend to ponder more about how things could have gone better or how things could have gone worse?”; and, (c) “When you look back at this experience, does it make more sense to you to reflect on how the outcome could have been better or how the outcome could have been worse?” Ratings were on 5-point scales anchored by [-2] = *Definitely Worse* to [2] = *Definitely Better*. We averaged these three items to create an index of counterfactual direction (α = .89).

Participants next completed the measure of optimism (LOT-R; [47]), which comprised 10-items, of which four were filler items, (e.g., “In uncertain times, I usually expect the best”; “I'm always optimistic about my future.”) that used 5-point agree-disagree scales. Ratings were averaged to create the optimism index (α = .83).

Finally, participants responded to demographic measures (age, gender, race and ethnicity) and an open-ended prompt about their interpretation of the study (“In the space below, please feel free to share any thoughts you have about this survey with us [optional]”) before they were thanked and paid for their participation. All studies reported here assessed the same demographic variables but because they explained no unique variation, we do not report on them further.

**Results and Discussion**

The counterfactual literature reports that upward counterfactuals outnumber downward counterfactuals in participant self-reports, and we replicated this finding using our 3-item scale measure. Specifically, with the continuous scale measuring counterfactual direction of comparison, the mean is greater than the midpoint (*M* = .23, *SD* = 1.28 vs. midpoint = 0; *t*(196) = 2.53, *p* = .012). If the scale is dichotomized at the scale midpoint, 45.2% of counterfactuals were upward and 39.6% were downward (the remaining 15.2% of counterfactuals fell at the scale midpoint of 0, and thus were neither upward nor downward). This lends confidence to our measure.

We next conducted our main analysis and regressed outcome valence (positive = 1; negative = 0), mean-centered optimism (*M* = 2.36, SD = 0.81), and their interaction on the index of counterfactual direction, which revealed an overall significant model, RMSE = 1.12, *p* < .001. Replicating prior work, this analysis revealed a main effect of outcome valence such that focusing on negative outcomes resulted in a greater tendency to generate upward (vs. downward) counterfactuals (*M* = 0.87, SD = 1.13) compared to focusing on positive outcomes (*M* = -0.35, SD = 1.12; *b* = -1.23, *SE* = .16, β = -0.48, *t*(193) = -7.67, *p* < .0001, 95% CI, [-1.54, -0.91]). We further noted no main effect of optimism (*b* = 0.11, *SE* = .15, β = 0.07, *t*(193) =0.74, *p* = .46, 95% CI [-0.19, 0.41]). Finally, the interaction between outcome valence and optimism was also not significant (*b* = 0.10 , SE = .20, β = 0.05, *t*(193) = -0.49, *p* = .62, 95% CI [-0.30, 0.49]).

**Study P2**

Study P2 was intended to be a replication of Study P1 in testing the relation between optimism and counterfactual direction of comparison, but with procedural modifications aimed at increasing the robustness of the Study P1 results. First, outcome valence was manipulated on a within-subject, rather than between-subject basis, which increased the statistical power to test interactions involving this variable. Second, the sample size was doubled. Third, and most importantly, we measured counterfactual direction in a different manner than in Study P1.

Specifically, Study P1 used a continuous, bipolar rating that captured both the specific counterfactual that participants reported, but also broader self-inferences about the general tendency to focus on upward versus downward counterfactuals across varying experiences. Although the three ratings varied in scope, they turned out to be highly intercorrelated. In Study P2 we tightened the measure of counterfactual direction to focus only on the specific counterfactual reported by participants, such that participants themselves classified that counterfactual as either upward or downward (i.e., a dichotomous rather than continuous measure). Other than these changes, Study P2 was identical to Study P1.

**Method**

Five hundred and one adults drawn from MTurk participated in our study (38% female; *M*_age_ = 37.4, SD_age_ = 12.8). Based on the exclusion criterion described in Study P1, we excluded seven participants, leaving a final sample of *N* = 494. The study ran from 3-9-2017 to 3-17-2017.

The study employed a 2 (outcome valence: positive vs. negative) × continuous (optimism) mixed design, whereby the first factor was within-subject and the second factor was between-subject. Upon entering the study, all participants read the instructions, “This survey asks you about TWO events from your recent past. Your job is to answer brief questions about what you remember.” Following this, outcome valence was manipulated by presenting the following in randomized order: “Think back to a recent POSITIVE [NEGATIVE] experience. In the space below, please share a few details about this experience” with order of outcome valence counterbalanced. We elicited counterfactuals in the same way as in Study P1. For the measure of counterfactual direction, participants answered: “Does your ‘what if’ thought focus on how things could have gone better or how things could have gone worse?” with a dichotomous response option. Optimism was again measured using the LOT-R (α = .89). Finally, participants provided demographic information (age, gender, race and ethnicity), then were thanked and paid for participation.

**Results and Discussion**

We again noted that upward counterfactuals outnumber downward counterfactuals with the current dichotomous measure (upward = 66.4%; downward = 33.6%, χ^2^(1, 978) = 106.7, *p* < .0001), which again lends confidence in our measure.

For our main analysis, we used R to conduct a logistic regression with our within-subject factor of outcome valence (positive vs. negative), our continuous between-subject measure of mean-centered optimism (*M* = 2.26, SD = 0.89), and their interaction, predicting whether the participant identified their counterfactual as upward (coded as 1) or downward (coded as 0). This analysis revealed a main effect of outcome valence (β = -1.75, *SE* = .43, χ^2^(1, *N* = 494) = -4.07, *p* < .001), such that negative (vs. positive) outcomes elicited more upward (vs. downward) counterfactuals. This analysis revealed no effect of optimism (β = 0.15, *SE* = .14, χ^2^(1, *N* = 494) = 1.06, *p* = .29), and the interaction between outcome valence and optimism was not significant (β = -0.11, *SE* = .18, χ^2^(1, *N* = 494) = -0.64, *p* = .52).

Study P2 was similar to Study P1 in showing no relation between optimism and downward counterfactuals, and again the failure to replicate the extant literature’s conclusion of a relation between optimism and downward counterfactual thinking.

**Study P3**

Study P3 further tested the relation between optimism and counterfactual direction of comparison, additionally testing whether coping judgments relate to counterfactual direction of comparison. As we noted in the introduction, upward counterfactual thinking has been shown to connect to performance improvement goals, whereas downward counterfactual thinking connects to affect regulatory goals. Coping judgments may follow this same rough distinction, with self-report measures of active coping (e.g., taking overt action to improve a situation) falling on the performance goal side, whereas positive reframing (e.g., rationalizing the situation to see it more positively) and denial (e.g., refusing to acknowledge the situation), fall more on the affect regulatory side. We tested again whether optimism predicts upward counterfactual thinking, and also whether this positive relation would be partly explained by the positive relation of active coping to upward counterfactuals, and/or by the negative relation between positive reframing or denial to upward counterfactual thinking (or the inverse, i.e., their positive relation to downward counterfactual thinking).

We used a similar design as in previous studies, but simplified the procedure such that participants recalled only a negative and not a positive experience, with optimism measured in the same way. Participants also made coping judgments along with judgments about their own counterfactual direction of comparison.

**Method**

Two hundred adults drawn from MTurk participated in our study (56% female; *M*_age_ = 37, SD_age_ = 11.0). Based on the same exclusion criterion as our previous studies, one participant was excluded, leaving a final sample of *N* = 199. The study ran on 4-4-2017.

Instructions were the same as in the previous studies, except that the outcome valence manipulation was reduced to only the negative outcome condition. Counterfactuals were elicited as before. Next, participants responded to 6 items about general patterns of coping (2 each assessing active coping, positive reframing, and denial) taken from Carver’s [64] Brief COPE scale. Participants received the instructions, “The questions below concern your negative experience: [participant’s experience piped here]. Please answer each question below using the scale provided” with endpoints anchored at [1] = *I haven’t been doing this at all* to [5] = *I’ve been doing this a lot*. Participants responded to two questions assessing their active coping: “I concentrated my efforts on doing something about the negative situation” and “I took action to try to make the situation better” (*r* = .66; *p* < .0001). Participants responded to two questions assessing their positive reframing: “I tried to see it in a different light, to make it seem more positive” and “I looked for something good in what happened” (*r* = .66; *p* < .0001). Participants responded to two questions assessing their denial: “I said to myself ‘this isn't real’” and “I refused to believe that it was happening” (*r* = .60; *p* < .0001). These six items were presented in random order. Counterfactual direction of comparison was assessed using the same 3-item measure as in Study P1 (α = .87) and optimism was measured using the LOT-R (α = .87).

Finally, participants provided demographic information (age, gender, race and ethnicity) and an open-ended prompt (“In the space below, please feel free to share any thoughts you have about this survey with us (optional)”) before they were thanked and paid for their participation.

**Results and Discussion**

We again noted that upward counterfactuals outnumber downward counterfactuals with the 3-item scale measure. As in Study P1, the mean is greater than the midpoint in the expected direction (*M* = 0.95, *SD* = 1.09 vs. midpoint = 0; *t*(198) = 12.34, *p* < .0001). If the scale is dichotomized at the scale midpoint, 70.9% of counterfactuals were upward and 17.1% were downward (the remaining 12.0% fell at the scale midpoint, thus were neither upward nor downward).

For our main analysis, we conducted a multiple regression with mean centered optimism (*M* = 2.55, *SD* = 0.83) along with the 3 coping measures (active coping, positive reframing, and denial) as independent factors and the index of counterfactual direction as the dependent measure, which showed an overall significant model, RMSE = 1.03, *p* < .001. Optimism was not meaningfully related to counterfactual direction of comparison (*b* = 0.02, *SE* = .09, β = 0.02, *t*(194) = 0.26, *p* = .80, 95% CI [-0.16, 0.21]). Further, active coping was not associated with counterfactual direction (*b* = 0.10, SE = 0.07, β = 0.10, *t*(194) = 1.48, *p* = .14, 95% CI [-0.03, 0.23]), but positive reframing predicted downward counterfactuals (*b* = -0.21, *SE* = .07, β = -0.24, *t*(194) = -3.20, *p* = .002, 95% CI [-0.34, -0.08]), as did denial (*b* = -0.24, *SE* = .07, β = -0.24, *t*(194) = -3.47, *p* < .001, 95% CI [-0.38, -0.10]). This study therefore also showed no relation between optimism and counterfactual direction.

**Study P4**

Study P4 was a direct replication of Study P1 that reinstated the between-subject manipulation of outcome valence and used the 3-item measure of counterfactual direction.

We also added a measure of event repeatability as an indirect indicator of the role of performance improvement goals. That is, Markman and colleagues [40] showed that a manipulation of event repeatability influenced counterfactual direction, such that repeating events evoked greater upward counterfactuals, presumably because such counterfactuals more usefully inform improvement on an ongoing rather than completed task. We measured rather than manipulated event repeatability, thus permitting a test of whether previously observed patterns are qualified by event repeatability, which might indirectly capture variation in performance goals.

**Method**

Three hundred and two adults drawn from MTurk participated in our study (52% female; *M*_age_ = 37, SD_age_ = 12). Using the prior exclusion criterion, we excluded 12 participants, leaving a final sample of *N* = 290. The study ran from 5-4-2017 to 5-6-2017.

The study design was the same as Study P1: 2 (outcome valence: positive vs. negative) × continuous (optimism) between-subject. Participants responded to the same 3-item measure as in Study P1 to assess counterfactual direction (α = .81) and optimism was again measured with the LOT-R (α = .80).

In addition, participants answered a question about repeatability, “Will the experience you described repeat in the future?” [1] = *yes* or [2] = *no*. Finally, participants responded to demographic measures (age, gender, race and ethnicity) and an open-ended prompt (“In the space below, please feel free to share any thoughts you have about this survey with us (optional)”) before being thanked and paid for their participation.

**Results and Discussion**

We again noted that upward counterfactuals outnumbered downward counterfactuals. As in Study P1, the mean was greater than the midpoint in the expected direction (*M* = .65 vs. midpoint = 0; *t*(289) = 9.50, *p* < .0001). If the scale is dichotomized at the scale midpoint, 65.9% of counterfactuals were upward and 27.6% were downward.

For our main analysis, we regressed outcome valence (positive = 1; negative = 0), mean-centered optimism (*M* = 2.43, SD = 0.76), and their interaction on index of counterfactual direction, and the overall model was significant, RMSE = 1.11, *p* < .001. As in Studies P1 and P2, this analysis revealed a main effect of outcome valence, such that negative outcomes elicited greater upward (vs. downward) counterfactual thinking (*M* = 1.03, SD = 1.05) than did positive outcomes (*M* = 0.29, SD = 1.17; *b* = -0.38, *SE* = .06, β = -0.33, *t*(286) = -5.89, *p* < .0001, 95% CI [-0.51, -0.26]). Our analysis also revealed a main effect of optimism (*b* = 0.15, *SE* = .09, β = 0.10, *t*(286) = 1.72, *p* = .09, 95% CI [-0.02, 0.32]), such that higher optimism was associated with more upward (vs. downward) counterfactuals. Finally, this analysis revealed a significant interaction between these factors (*b* = 0.15, SE = .09, β = 0.10, *t*(286) = 1.78, *p* = .08, 95% CI [-0.02, 0.32]).

We used Model 1 of the PROCESS macro for SPSS [65] to further probe the nature of this interaction, which showed that, given positive outcomes, higher optimism was related to the generation of more upward (vs. downward) counterfactuals (*b* = .30, SE = .12, *t*(286) = 2.46, *p* = .01); however, given negative outcomes, this effect was attenuated and optimism was unrelated to counterfactual direction (*b* = -.01, SE = .12, *t*(286) = -.04, *p* = .97).

Finally, as might be expected, outcome valence was related to optimism (*b* = -0.07, SE = .04, *t*(288) = -1.63, *p* = .104), such that recalling positive events generated greater optimism (*M* = 2.50) than recalling negative events (*M* = 2.36). This finding suggests that measuring optimism and manipulating event recall may be better suited to experiments using two different time periods so as to avoid any unintended effects of the counterfactual elicitation on the independent measure of trait optimism. No effects involving event repeatability were significant.

Thus, Study P4, again provided evidence that optimism is not related to downward counterfactual thinking; but rather showed evidence of a weak positive relation between optimism and upward counterfactual thinking.

**Study P5**

Although the previous studies indicate that optimism is not associated with downward counterfactual thinking, we have yet to observe any variability in this effect that might account for the discrepancy between the current findings and previously published findings. One possible source of variability is the scale used to measure individual differences in optimism. Thus far we have employed the LOT-R, but several earlier studies that showed that optimism predicts downward counterfactual thinking used the DPQ [7, 10, 11]. Prior theorists have assumed that the LOT-R measures a broad, general self-inference of beliefs regarding the future, whereas the DPQ captures more tactical aspects of thought frequency, concern, and worry about particular outcomes. We wondered whether one aspect that differentiates the current results versus those of prior studies is the choice of scale measure of optimism. That is, the LOT-R might reveal a relation between optimism and upward counterfactual thinking whereas the DPQ might reveal a relation between optimism and downward counterfactual thinking. Accordingly, the main goal of Study P5 was to compare directly the LOT-R against the DPQ in their relation to counterfactual direction of comparison.

Of key concern was which to use of the versions available of the DPQ (e.g., the original version in [52]). Sanna [10, 11] used an 8-item version of the original of the DPQ, but with the 4 items assessing defensive pessimism subtracted from the 4 items assessing optimism. The revised 12-item version is specified by averaging across all items with appropriate reverse scoring, yet contains two correlated subscales of pessimism and reflectivity (which nevertheless both load substantially on a single unrotated factor). In deciding how best to test the DPQ, we tried both to capture what had been done in earlier studies while adhering to currently accepted analytic standards and revised measurement tools in optimism. Thus, to ensure that we had DPQ items shared with Sanna [10, 11], we identified the shared 4-item subset of the DPQ-R, averaging them to create the DPQ index of optimism.

**Method**

Two hundred students drawn from the undergraduate population at a Midwestern U.S. university participated in our study (58% female; *M*_age_ = 21, SD_age_ = 4.2). Based on the same exclusion criterion as our previous studies, two participants were excluded from our analysis and two students dropped out and their data had to be removed from analyses (*N* = 196). The study ran from 10-3-2017 to 10-5-2017.

The study design was the same as Study P1 except that optimism was measured in 2 ways, with the six items of the LOT-R (α = .79) and the four items from the Revised DPQ (α = .68), with order of presentation counterbalanced. As we would expect, these scales were correlated (*r* = .59, *p* < .0001). Finally, participants responded to demographic measures (age, gender, race and ethnicity) and an open-ended prompt (“In the space below, please feel free to share any thoughts you have about this survey with us (optional)”) before being thanked and paid for their participation.

**Results and Discussion**

We again noted that upward counterfactuals outnumber downward counterfactuals with the 3-item scale measure. As in Study P1, the mean is greater than the midpoint in the expected direction (*M* = .64 vs. midpoint = 0; *t*(195) = 8.36, *p* < .0001). If the scale is dichotomized at the scale midpoint, 60.2% of counterfactuals were upward and 23.5% were downward.

We therefore proceeded with our main analysis and regressed outcome valence (positive = 1; negative = 0), mean-centered LOT-R (*M* = 2.26, *SD* = 0.68), mean-centered DPQ (*M* = 3.98, SD = 1.15), the interaction of outcome valence × LOT-R, and the interaction of outcome valence × DPQ on the index of counterfactual direction, which showed an overall significant model, RMSE = 0.87, *p* < .001. This analysis revealed a main effect of outcome valence, such that negative outcomes elicited greater upward (vs. downward) counterfactual thinking (*M* = 1.25, SD = 0.65) than did positive outcomes (*M* = -0.003, SD = 1.05; *b* = -1.25, SE = .13, β = -0.59, *t*(190) = -9.94, *p* < .001, 95% CI [-1.50, -1.00]).

We also noted no effect of LOT-R, (*b* = 0.11, SE = .14, β = 0.07, *t*(190) = 0.79, *p* = .43, 95% CI [-0.17, 0.40]), or DPQ, (*b* = -0.06, SE = .09, β = -0.07, *t*(190) = -0.72, *p* = .47, 95% CI [-0.23, 0.11]). Finally, there were no significant interactions, either between LOT-R and valence (*p* = .38) or between DPQ and valence (*p* = .43).

In probing the above relations further, we noted that outcome valence in this study influenced responding on both the LOT-R and the DPQ, as would not be expected if the measures capture situationally-stable individual differences. Those in the negative outcome condition gave higher optimism (*M* = .08) ratings on the LOT-R versus those in the positive outcome condition (*M* = -0.09; *t*(194) = -1.71, *p* = .09); and the same was the case for the DPQ (*M*s = 0.17 vs. -0.20; *t*(194) = -2.24, *p* = .03). The procedural aspect that the individual difference questions came after the outcome valence manipulation was common to all of our previous studies (save Study P3, which had no such manipulation, but which did place the counterfactual solicitation ahead of the individual difference measurement). To resolve this ambiguity, and to more closely replicate prior works that elicited optimism in mass testing sessions prior to counterfactual elicitation, the ideal study design would separate temporally the participants’ responses to these two measures. This was the rationale for our final preliminary study.

**Study P6**

Study P6 was pre-registered on 3-3-2018. In Study P5, we noted that both measures of optimism varied as a function of outcome valence. Accordingly, to remove possible order effects in responding, Study P6 dispersed the optimism and counterfactual measures to two time points separated by one week.

An a priori power analysis was conducted using G*Power v3.1.9.4 to determine the minimum sample size required to find significance with a desired level of power set at .80, an alpha (α) level at .05, and weak effect sizes for the following planned analyses: a linear regression and the individual path coefficient t-tests for the predictors in that model. Based on this analysis, it was determined that a minimum of 314 participants were required to ensure adequate power for the simple effect of the linear regression (namely, our two measures of optimism: LOT-R and Revised DPQ). From our prior experience with attrition rates, we assumed a completion rate of .6 from time-1 (T1) to time-2 (T20) and on this basis we set the desired sample size for T1 at *N* = 523, which we increased by 50% and rounded up, to be conservative, for a final target sample size of *N* = 800 for the T1 measurement.

For increased robustness of the counterfactual measure, each participant generated a total of four counterfactuals; two in response to a positive and two in response to a negative recalled outcome.

**Method**

Eight hundred and two adults from MTurk (52% female; *M*_age_ = 37.4, SD_age_ = 11.2) responded at time-1 to scale questions measuring optimism and self-esteem. Of these participants, 537 returned for the time-2 measurement (67% return rate; 54% female; *M*_age_ = 38.0, SD_age_ = 11.3). Based on the exclusion criterion used in the previous studies, the first author determined 12 participants had not followed the instructions of the prompt, leaving *N* = 525. Time-1 measurement was on 3-5-18 and time-2 measurement ran from 3-12-2018 to 3-14-2018.

At time-1, participants responded to three scales—the LOT-R, the Revised DPQ, and Rosenberg’s self-esteem scale [66], the latter scale added to clarify the validity of the optimism measures, with all items presented in random order and using the same scale designations (anchored by 1 = *Not at all* to 7 = *Extremely*). We averaged the six LOT-R items into one index of optimism (α = .91), the four items from the revised DPQ into a second index of optimism (α = .80), and the 10 self-esteem items (α = .94). The LOT-R and revised DPQ optimism scales were highly correlated (*r* = .78, *p* < .0001), and each scale correlated with self-esteem (*r* = .80, *p* < .0001 and *r* = .78, *p* < .0001, respectively). Finally, participants reported demographic information (age, gender, race and ethnicity).

One week later, participants were invited to the time-2 session. The invitation did not reference the time-1 survey nor give any indication that the two surveys were related. The survey began with the instructions, “This survey asks you about four separate events from your recent past. Your job is to answer brief questions about what you remember.” Participants were randomly assigned to order of outcome valence, either first recalling two positive events then two negative events, or the reverse order.

Participants read the same prompts eliciting outcome recall and counterfactual thinking as in the previous studies. Participants then responded to the same dichotomous measure of counterfactual direction as in Study P2, in which they indicated whether the specific counterfactual was upward or downward. We summed across these questions to gain a total count of upward and downward counterfactuals. (Note that with this procedure, the numbers of upward versus downward counterfactuals are perfect inverses, always summing to 4).

After indicating the direction of their counterfactual, participants next rated the counterfactual in terms of its connection to performance goals (“Thinking about this alternative makes me want to improve my performance in the future”; rated on 7-point agree-disagree scale) and affect regulatory goals (“Thinking about this alternative makes me feel …”; rated on 7-point better-worse scale). Finally, participants answered demographic questions (age, gender, race and ethnicity).

**Results and Discussion**

We first regressed mean centered LOT-R optimism (*M* = 4.72, SD = 1.44) on our index of upward counterfactuals (recall that number of downward counterfactuals is the perfect inverse of upward counterfactuals), which revealed that optimism trended positively with upward counterfactuals (*b* = .04, *SE* = .03, β = 0.06, *t*(523) = 1.43, *p* = .15, 95% CI [-0.02, 0.11]). Second, we regressed mean centered DPQ optimism (*M* = 4.50, *SD* = 1.40) on our index of upward counterfactuals; and, DPQ optimism trended positively with upward counterfactuals (*b* = .04, *SE* = .03, β = 0.06, *t*(523) = 1.38, *p* = .17, 95% CI [-0.02, 0.11]).

Study P6 added to the previous studies in that, even with a large, pre-registered sample in which measurement of individual difference variables was temporally removed from measurement of counterfactual thinking, optimism was still unrelated to counterfactual direction of comparison. Further, we failed to account for the discrepancy between current and prior findings in the literature due to the use of different scales to capture optimism.

**References**

1. Carver CS. You want to measure coping but your protocol's too long: Consider the brief COPE. Intl J Behav Med 1997;4(1):92-100. doi: 10.1207/s15327558ijbm0401_6
2. Hayes AF. Introduction to mediation, moderation, and conditional process analysis. New York: Guilford Press; 2013.
3. Rosenberg, MJ. Society and the adolescent self-image. Princeton: Princeton University Press; 1965.
